# Supplementary material for: Sestrin2 Expression Has Regulatory Properties and Prognostic Value in Lung Cancer
Source: J Pers Med. 2020 Sep 1;10(3):109. doi: 10.3390/jpm10030109 (PMC7565522; doi:10.3390/jpm10030109)

Supplement figure S1. Expression of Sestrin2 (SESN2) in various cell lines measured by RT-PCR.

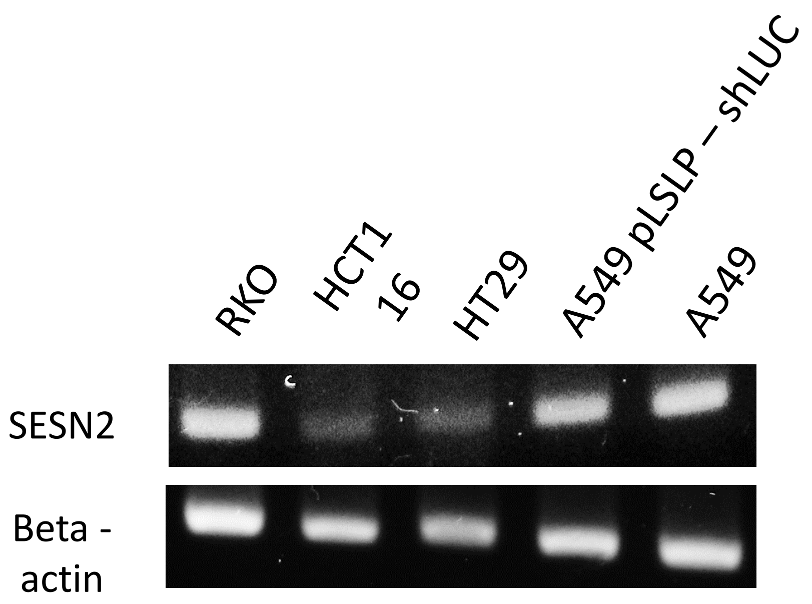

Supplement figure S2. Cell Proliferation assay of scramble, shshSESN2-1, and shSESN2-2 cells. Cell viability was measured using EZ-cytox reagent at 1,2,3, and 4 day after seeing and incubating cells ($1X10^4$ per well in 96-well plate). The absorbance was measured at 450 nm using a fluorescence microplate reader

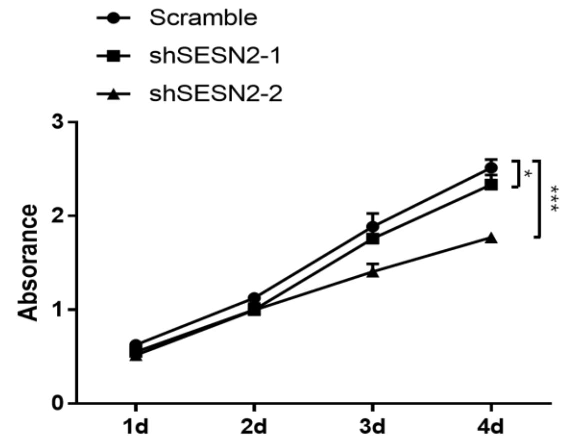

Supplement figure S3. Expression of NRF2 and HO1 by RT-PCR in knockdown A549 lung cancer cell.

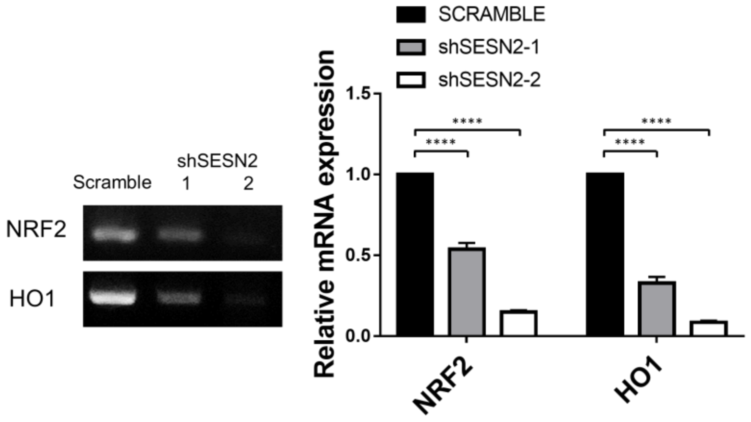

Supplement: Supplementary file 1 [file jpm-10-00109-s001.pdf]
